# New insights into pathogenisis and therapies of P2X7R in Parkinson’s disease

**DOI:** 10.1038/s41531-025-00980-7

**Published:** 2025-05-05

**Authors:** Shizhuang Wei, Xiaoyu Song, Yakui Mou, Ting Yang, Yao Wang, Hanrui Wang, Chao Ren, Xicheng Song

**Affiliations:** 1 Department of Otorhinolaryngology, Head and Neck Surgery, Yantai Yuhuangding Hospital, Qingdao University, Yantai, China; 2 Shandong Provincial Key Laboratory of Neuroimmune Interaction and Regulation, Yantai Yuhuangding Hospital, Yantai, China; 3 Shandong Provincial Clinical Research Center for Otorhinolaryngologic Diseases, Yantai Yuhuangding Hospital, Yantai, China; 4 Yantai Key Laboratory of Otorhinolaryngologic Diseases, Yantai Yuhuangding Hospital, Qingdao University, Yantai, China; 5 Department of Neurology, Yantai Yuhuangding Hospital, Qingdao University, Yantai, China

**Keywords:** Diseases of the nervous system, Neuro-vascular interactions, Neuroimmunology

## Abstract

Parkinson’s disease (PD), a prevalent neurodegenerative disorder, is linked to genetics and environment, but its mechanisms remain unclear. Emerging evidence connects purinergic signaling-particularly ATP-sensitive P2X7 receptor (P2X7R)-to PD. P2X7R expression is elevated in PD patients, and its antagonist BBG mitigates 6-OHDA-induced dopaminergic neuron death. This review discusses P2X7R’s structure, neural functions, PD-related mechanisms, and therapeutic potential as a targert.

## Introduction

Parkinson’s disease (PD) is the second most prevalent neurodegenerative disorder, following Alzheimer’s disease, and affects approximately 1% to 2% of the elderly population^1^. The clinical features of PD primarily include motor symptoms, *eg*. resting tremor, rigidity, and bradykinesia, along with non-motor symptoms such as olfactory dysfunction, sleep disturbances, pain, and emotional and psychiatric issues. These symptoms significantly impair patients’ quality of life and reduce the effectiveness of treatments^2,3^. Pathologically, PD is characterized by the gradual loss of dopaminergic neurons in the substantia nigra and the formation of Lewy bodies^4^. Since the identification of PD more than 200 years ago^5^, research has established that multiple factors, including environmental, genetic, and aging factors, contribute to the onset of the disease^6^. However, the specific mechanisms involved in PD remain largely undetermined.

In terms of clinical treatment for PD, the primary pathological characteristic is the death of dopaminergic neurons, which leads to a reduction in dopamine production. Therefore, treatments for PD rely mainly on the use of the large-dose dopamine precursor levodopa for dopamine replacement therapy, as well as dopamine receptor agonists^7–9^. L-DOPA can cross the blood-brain barrier and is converted into dopamine, which binds to specific subtypes of dopamine receptors (D1R-D5R)^8^. However, long-term use of L-DOPA can lead to an imbalance in the motor system’s striatal circuits and cause side effects, such as L-DOPA-induced dyskinesia and motor complications. After five years of continuous treatment, approximately 50% of patients experience side effects, including motor complications and tremors^10–12^. Additionally, prolonged use of dopamine receptor agonists may induce impulse control disorders and sleep disturbances^13,14^. Managing and treating these complications is challenging, making it necessary to develop new effective therapies to address motor fluctuations and movement disorders.

P2X7R is a member of the P2X family, which consists of ATP-sensitive cation channel receptors. These receptors are widely present in various tissues of mice, rats, humans, and other organisms^15^. P2X7R is expressed in multiple cell types throughout both the peripheral and central nervous systems. Specifically, P2X7R is highly expressed in microglia, where it promotes oxidative stress by inducing calcium influx and regulating protein kinases, NADPH oxidase, and mitochondrial activity in the extracellular environment. Additionally, P2X7R is expressed in neurons and astrocytes^16,17^. As research has advanced, P2X7R has been identified as a critical molecule in the central nervous system, particularly in that of the Alzheimer’s disease patients^18,19^. However, the specific role of P2X7R in PD has not been extensively explored. In this review, we will provide a comprehensive overview, beginning with a brief introduction to P2X7R and purinergic signaling. We will then highlight the molecular structure and functions of P2X7R, emphasizing its roles and potential mechanisms in PD. Finally, we will discuss the potential therapeutic implications of P2X7R-targeted therapy in the treatment of PD.

## Purinergic Signaling and P2X7R

In the human body, adenosine triphosphate (ATP) not only serves as an intracellular energy source but also functions as an extracellular signaling molecule that mediates intercellular communication and interactions between neurons and glial cells^20,21^. In 1978, Burnstock introduced the concept of “purinergic receptors,” which are classified into two main types: P1 receptors, which are activated by adenosine, and P2 receptors, which respond to ATP and its analogs^22–24^. The purinergic signaling system comprises enzymes, transporters, and receptors, including the ionotropic P2X and metabotropic P2Y receptor families, which are responsible for the synthesis, release, signal transduction, and inactivation of extracellular ATP and its metabolites, such as adenosine^23^.

Four subtypes of P1 receptors have been cloned: A1, A2A, A2B, and A3. The A1 and A3 receptors primarily couple with the Gi proteins, inhibiting adenylate cyclase, whereas the A2A and A2B receptors couple with the Gs and Go proteins to stimulate the production of cyclic AMP (cAMP). P2 receptors are categorized into P2X and P2Y. Currently, seven P2X subtypes (P2X1 − 7) and eight mammalian P2Y receptor subtypes (P2Y1,2,4,6,11,12,13,14) have been cloned^25^. These receptors play functional roles throughout the body, including in the brain, where they have been demonstrated to be involved in various cellular processes, such as neurotransmission, glial cell communication, neuromodulation, cell survival, proliferation, differentiation, and neuroinflammation^26^.

The purinergic P2X7R is part of the ionotropic P2X receptor family, which comprises seven members designated P2X1R through P2X7R. P2X7R was first cloned in 1996^27^. Notably, several splice isoforms of P2X7R have been identified, distinguishing it from other members of the P2X receptor family^28^. In humans, only the A and B isoforms result in functional receptors. P2X7A is the full-length isoform, whereas P2X7B is a truncated variant. Although the B isoform can form a functional channel, it is incapable of forming a membrane pathway called the “large pores”^29^.

Like other P2X receptor types, P2X7R assembles into a trimeric structure composed of three identical subunits^30,31^. Each subunit contains N- and C-terminal regions, two transmembrane domains, and a large extracellular loop. The agonist binding sites are located at the interface between two adjacent subunits^32,33^. Interestingly, unlike other members of the P2X receptor family, P2X7R does not form heterodimers with other P2X family members, except with P2X4R^34^.

In addition to being a typical cation-selective channel similar to other P2X receptors, P2X7R is notable for its ability to activate the large pore, which permits the uptake of extracellular molecules up to 900 Da^35,36^. Initially, it was believed that the large pore formed only after prolonged stimulation and was extrinsic to P2X7R, requiring auxiliary protein recruitment. It was thought to mediate most of the P2X7R-dependent functions. However, recent findings now suggest that the large pore is intrinsic to P2X7R and is activated immediately following receptor stimulation^37,38^. Reports indicate that P2X2R and P2X4R can also take up hydrophilic organic molecules with molecular weights of approximately 400 Da^39^; however, P2X7R has been characterized more reliably in this context and shows the greatest reproducibility.

## Structure of P2X7R

**Fig. 1:**
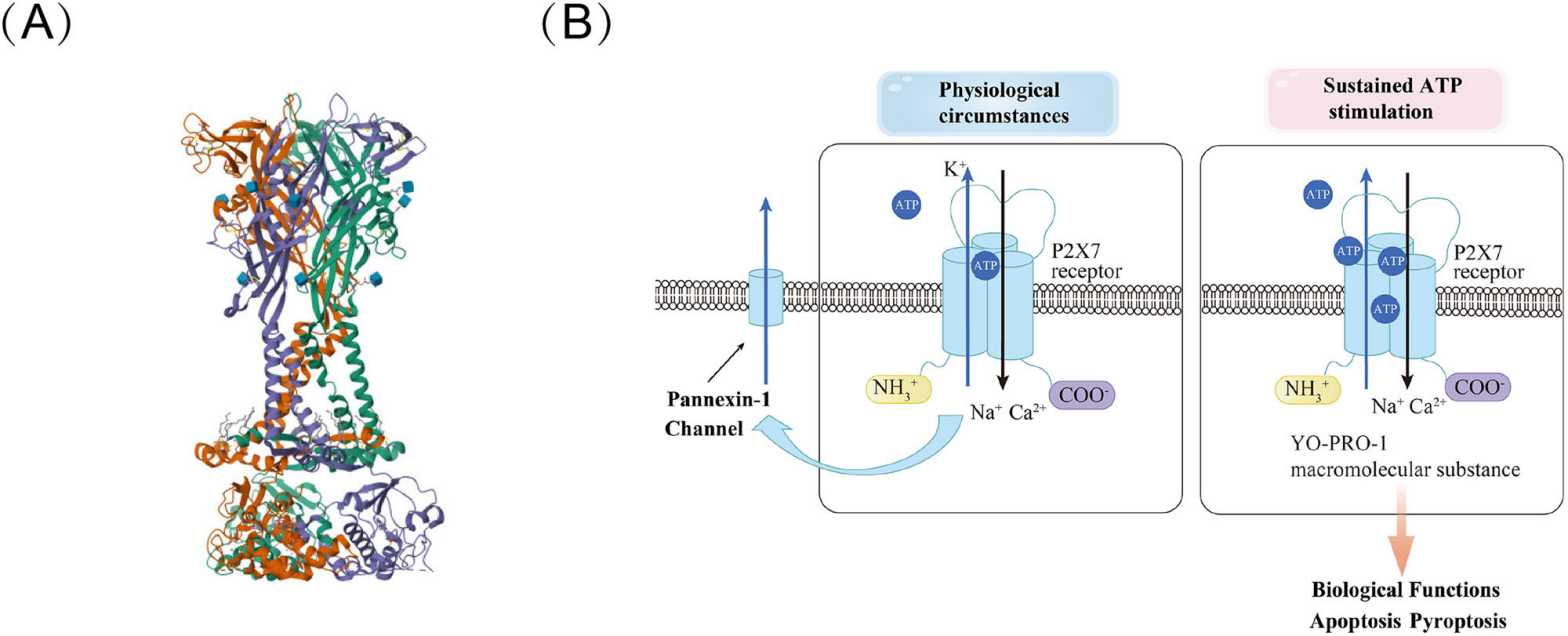
Molecular structure of P2X7R. **A** Schematic illustration of the molecular structure of P2X7R and its subunits. The crystal structure of a single subunit of *Ailuropoda melanoleuca* P2X7R is depicted from the RCSB database (https://www.rcsb.org). The three identical subunits are represented in green, orange-yellow, and purple, with each subunit featuring unique binding sites for various molecules. **B** Structural changes of P2X7R under different circumstances. Under physiological conditions, the binding of ATP to P2X7 facilitates K^+^ efflux and the influx of Ca^2+^ and Na^+^. Furthermore, P2X7R connects with Pannexin-1 to mediate ATP release. Prolonged ATP stimulation results in the formation of a large molecular channel that permits the passage of many molecules, such as YO-PRO1, thus carrying out biological functions.

In human, the coding gene of P2X7R locates on the long arm of chromosome 12, specifically at 12q24.31, near the *P2X4R* locating at 12q24.32. In contrast, both mouse *P2x7r* and *P2x4r* are found on chromosome 5 at positions 62.50 and 62.43 cM, respectively. The proximity of *P2X4R* and *P2X7R*, along with their protein sequence homology, suggests that these receptors originated from gene duplication^40^. P2X7R comprises 595 amino acids, featuring an N-terminal region (26 aa), a C-terminal region (239 aa), an intracellular domain, and two conserved transmembrane domains (TM1, TM2). Both the N- and C-termini are located intracellularly, forming a trimeric structure of homologous subunits in the cell membrane^41^ (Fig. 1). ATP can activate all subunits of the P2X receptor family, but there are significant differences in their affinities, and the rate of desensitization varies among different subunits. Unlike other ionotropic receptors in the P2X family, P2X7R does not desensitize to prolonged or repeated ATP stimulation. Instead, it exhibits two distinct changes^42,43^: first, with repetitive or extended application of the agonist, the current mediated by P2X7R gradually increases and it triggers a non-inactivating influx of Ca²⁺ and Na⁺ ions, along with the efflux of other ions, resulting in rapid depolarization^44,45^; second, continuous stimulation of P2X7R by ATP can lead to increased permeability and can induce the formation of a non-selective membrane pore that permits the passage of large hydrophilic molecules up to 900 Da, allowing large molecules to enter or exit the cell, including nanoscale molecules such as the fluorescent dye YO-PRO-1^36,46^. This can severely disrupt the intracellular ionic balance and potentially lead to cell death through mechanisms such as apoptosis, necrosis, or pyroptosis, further aggravating organ damage^47,48^.

## Role of P2X7R in the Central Nervous System

### P2X7R and Microglia

**Fig. 2:**
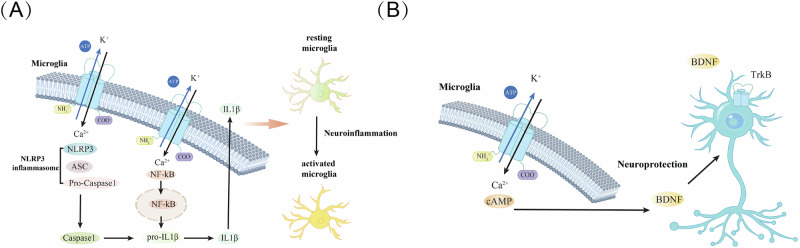
Role of P2X7R in microglia. **A** Neuroinflammation: In pathological conditions, when ATP binds to P2X7R on the surface of microglia, it can lead to an overload of intracellular Ca^2+^, which activates the assembly of inflammasomes. This activation facilitates the cleavage of pro-caspase-1 into caspase-1, leading to the further cleavage of pro-IL-1β into IL-1β, which is then released into the extracellular space. Additionally, this process activates the NF-κB pathway, resulting in the release of inflammatory factors such as IL-1β. These reactions cause microglia to transition from a resting state to an active state, thereby influencing nearby glial cells or neurons and promoting the neuroinflammatory response. **B** Neuroprotection: When ATP binds to P2X7R on microglia, it enhances the activity of intracellular transcription factors that affect the secretion of brain-derived neurotrophic factor (BDNF). This BDNF subsequently interacts with TRKB receptors on neuron surfaces, exerting neuroprotective effects.

Microglia serve as resident macrophages in the brain and play a vital role in maintaining the immune functions of brain and the first responders to pathological insults^49^. Since their discovery by PIO del Rio Hortega in 1919, research interest in their function within the central nervous system has progressively increased. Studies have indicated that microglia originate from yolk sac progenitors that invade the brain early in embryonic development^50,51^. Current research suggests that P2X7R is primarily expressed on microglia and that different P2X receptor subtypes are expressed at various developmental stages. Specifically, the P2X1 and P2X4 subunits are present in embryonic microglia, with approximately 30% of these cells expressing the P2X7 receptor. In contrast, in adult microglia, only the P2X4 and P2X7R subunits are expressed^52,53^. In the healthy central nervous system, P2X7R-expressing microglia are widespread across nearly all brain regions. Functionally, P2X7R on the microglia play different roles. Under inflammatory conditions, P2X7R mediates the activation of inflammasomes, the stimulation of microglial cell activity, and the release of interleukins and proinflammatory factors^54,55^. Notably, the activation of P2X7R on microglia also has neuroprotective effects, primarily via the activation of intracellular transcription factors such as the cAMP response element, which mediates various protective actions^56^ (Fig. 2). More significantly, elevated levels of P2X7R expression on microglia have been observed in neurological disorders such as Alzheimer’s disease, multiple sclerosis, meningitis, and Amyotrophic lateral sclerosis^57–60^, suggesting that P2X7R plays a crucial role in a range of neurological diseases.

### P2X7R and Astrocytes

**Fig. 3:**
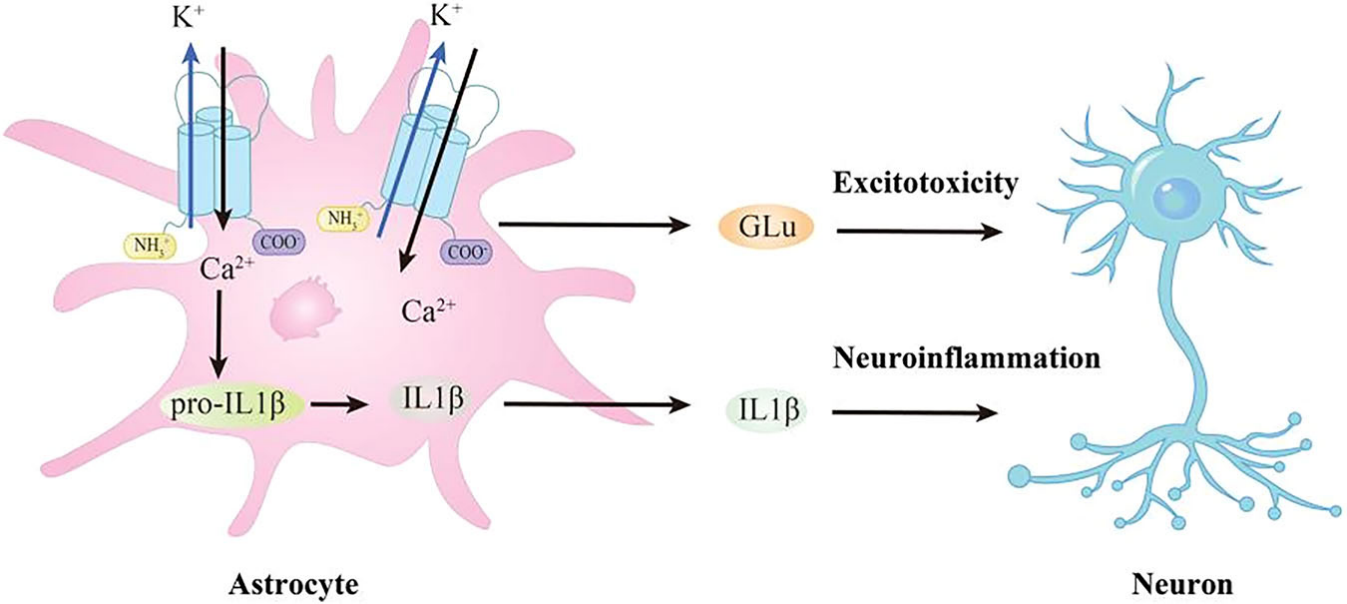
Role of P2X7R in astrocyte. Exogenous ATP binds to P2X7R on the surface of astrocytes, which can lead to the cleavage of pro-IL-1β into IL-1β via different pathways, thereby promoting the secretion of extracellular IL-1β and allowing astrocytes to play a regulatory role in neuroinflammation. Additionally, glutamate, which is predominantly produced by astrocytes, can be released through P2X7 receptors, exerting neurotoxic effects that result in neuronal damage and cell death.

Astrocytes are a subtype of glial cells that exhibit a star-like appearance when stained with Golgi’s method. As discovered by Michael Von Lenhossek in 1895, these cells are derived from the ectoderm and neuroepithelium and make up approximately 20% to 40% of the total number of central glial cells in humans^61^. Research has revealed that astrocytes comprise various subpopulations^62^. Studies focusing on P2X receptors in astrocytes have shown that all types of P2X receptor mRNAs and proteins are expressed, with P2X6 and P2X7 being detected in hippocampal astrocytes^63^. Functionally, astrocytic P2X7R has been shown to regulate the balance between neuronal excitation and inhibition by modulating the release of neurotransmitters such as glutamate and gamma-aminobutyric acid, facilitating crosstalk with neurons^64^. Additionally, under pathological conditions, increased ATP levels in the environment can activate P2X7R on astrocytes, promoting the processing and release of IL-1β and other pro-inflammatory factors^65^. Notably, activation of astrocytic P2X7R can lead to a neurotoxic phenotype, resulting in motor neuron death; however, the use of selective P2X7R inhibitors effectively reduces the expression of inflammatory factors^66^ (Fig. 3). These studies suggested that under both physiological and pathological conditions, astrocytic P2X7R plays a significant role through distinct modes of communication with neurons.

### P2X7 and Oligodendrocytes

Oligodendrocytes are a type of glial cell in the central nervous system, distinct from astrocytes and microglia. In terms of their distribution, oligodendrocytes are the primary component of white matter, which constitutes approximately 50% of the total central nervous system (CNS) in the human brain^67^. Functionally, oligodendrocytes serve as myelinating cells in the CNS, and they are subjected to cellular stress and subsequent death in various metabolic or inflammatory diseases. The presence of viable oligodendrocytes and intact myelin is essential for neuronal health. Myelination and remyelination processes continue into adulthood, with the synthesis of new myelin playing a role even during learning. The differentiation of new oligodendrocytes (OLs) from oligodendrocyte progenitor cells (OPCs) is an important part of this process^68^.

Regarding the expression of P2X7R in oligodendrocytes, there is evidence suggesting that oligodendrocytes and their progenitor cells express P2X7 receptors in vitro^69,70^. Under physiological conditions, purinergic receptors have also been shown to participate in the development of oligodendrocytes^69,71^. Purinergic signaling regulates the self - renewal of OPCs, and the P2X7 receptor subtype may be of potential importance in age - related changes in OPC metabolism^71^. P2X7R mediates the ATP-induced migration of oligodendrocyte progenitor cells^72^. Additionally, in hippocampal CA3 pyramidal neurons, the deletion of P2X7Rs in oligodendrocytes (NG2 glial cells) eliminates the Bz - ATP - induced current response^73^. Under pathological conditions, P2X7 is present in oligodendrocytes in the hippocampus, cortex, striatum, thalamus, and cerebellum after ischemia, traumatic injury, and status epilepticus. The abnormal increase in ATP continuously activates the P2X7 receptor, leading to oligodendrocyte damage and the occurrence of related diseases^74,75^. The above - mentioned research indicates that P2X7 mediates the functions of oligodendrocytes through different pathways.

### P2X7R and Neurons

While P2X7R has the highest localization density in microglia and a relatively lower density in glial cells, the expression of P2X7R in neurons remains controversial. This controversy largely stems from the selectivity of the antibodies used^76–78^. To explore the expression and function of P2X7R in neurons, researchers have evaluated P2X7R mRNA and protein levels, conducted electrophysiological recordings, and measured changes in intracellular calcium levels induced by ATP or Bz-ATP, both in the presence and absence of selective P2X7R antagonists or P2X7R-specific siRNAs. Current evidence supporting the expression of P2X7R in neurons can be highlighted in several ways. First, P2X7R mRNA and protein have been detected in various immortalized human and mouse cell lines. In neuronal cell lines, P2X7 antagonists effectively suppressed the increase in intracellular calcium level induced by ATP^79,80^. Additionally, pharmacological inhibition or knockdown of P2X7R promoted the differentiation of N2a cells by enhancing neurite outgrowth^81^. Functional P2X7R has also been identified in embryonic stem cells (ESCs) and neural progenitor cells (NPCs) derived from neuron-like human ESCs, as well as in the subventricular zone of adult mice and the striatum of developing embryos^82–84^. Importantly, P2X7R plays a crucial role in neuronal differentiation and physiology by regulating axonal growth and synaptic function^85,86^. Nevertheless, further exploration is needed to better understand the expression and function of P2X7R in neurons.

## P2X7R and Parkinson’s disease

Research has revealed that P2X7R is associated with the occurrence and development of Alzheimer’s disease, Huntington’s disease and Amyotrophic lateral sclerosis^57–60^, and the relationship between P2X7R and PD has also attracted attention. It is generally believed that genetic polymorphisms are associated with age, sex-related degenerative processes, and susceptibility differences. Clinical studies have shown that the *P2X7R* gene is highly polymorphic and has been identified as a susceptibility gene for various diseases^87–89^. In a clinical study focused on the Chinese population, the correlations between the polymorphisms 1513 A > C (rs3751143) and 1729 T > A (rs1653624) and the incidence of PD were compared among 285 sporadic PD patients and 285 healthy Han individuals. The research revealed a close correlation between the P2X7R polymorphism 1513 A > C and sporadic PD, early-onset PD, and male PD in the Chinese population^90^. However, when the P2X7R PET radioligand tracer [11 C]JNJ717 was used to examine the results in healthy volunteers and PD patients, no significant differences were observed between the two groups^91^. This may be related to different pathogenic mechanisms in PD patients. Notably, P2X7R expression is upregulated in PD patients^92^. Additionally, a prospective study revealed increased mRNA and protein expression of the P2X7R/NLRP3 inflammasome in the peripheral blood mononuclear cells of PD patients^93^, indicating that peripheral P2X7R may be associated with the early stages of the disease.

According to preclinical studies, P2X7R mRNA and protein levels are elevated in the striatum and substantia nigra of PD models induced by 6-OHDA and MPTP^94^. In the 6-OHDA-induced PD model, pre-injection of the P2X7R antagonist A-438079 (30 mg/kg, i.p.) prevented dopamine depletion in the striatum without reducing the death of dopaminergic neurons^95^. Additionally, these findings are consistent with results showing that chronic brilliant blue 250 (BBG) treatment (45 mg/kg, i.p.) prior to unilateral 6-OHDA injection prevented the death of tyrosine hydroxylase-positive neurons^96^. However, in MPTP- and rotenone-induced PD models, the absence of P2X7R did not reduce the death of dopaminergic neurons^94^. This might be related to the difference in PD models induced by various neurotoxins. In cell experiments, BBG (100 nM) attenuated 6-OHDA-induced neurotoxicity in SH-SY5Y cells^97^. Rotenone, a neurotoxin used to construct PD models, could promote the secretion of inflammatory factors by increasing the current density of P2X7 channels on astrocytes^98^. Interestingly, depression can exacerbate the occurrence of PD through microglial P2X7R-mediated neuroinflammation^99^. These studies suggested that P2X7R may serve as a target for the diagnosis and treatment of PD.

## Mechanisms Associated with P2X7R in the Development of PD

### P2X7R and Neuroinflammation

Clinical studies have confirmed that, alongside the significant loss of dopaminergic neurons, PD patients frequently exhibit abnormal activation of glial cells in the substantia nigra^100^. Neuroinflammation resulting from excessive glial cell activation is increasingly recognized as a key factor in the pathogenesis of PD. Microglia, as the primary responder in neuroinflammation, not only react to pathogen invasion and external injury but also play an essential role in maintaining homeostasis of the brain’s microenvironment as important immune cells while providing certain degree of neuroprotection^49^. However, when exposed to sustained stimuli, such as brain injury, microglia can become aberrantly activated, producing and releasing substantial amounts of pro-inflammatory factors, which leads to the inflammatory loss of neurons^101^. P2X7R, an ATP-sensitive ion channel, is expressed predominantly in microglia within the brain. P2X7R plays a crucial role in mediating the expression of pro-inflammatory cytokines from the IL-1 family, significantly contributing to the innate immune response. The activation of P2X7R can trigger multiple inflammatory signaling pathways, including the nuclear factor kappa-light-chain-enhancer of activated B cells (NF-κB) pathway. NF-κB is a vital transcription factor that regulates the expression of numerous inflammation-related genes. Additionally, the activation of P2X7R on microglia initiates the innate immune response by promoting the assembly of the NLRP3 inflammasome and the activation of caspase-1^102,103^. In acute PD models, increased P2X7R binding has been linked to neuroinflammation. Previous studies have also demonstrated that LPS injection into the striatum significantly increased P2X7R expression in microglia. In LPS-induced models of PD, P2X7R expression is upregulated^104^, and the P2X7 inhibitor BBG (50 mg/kg, i.p., 15 days) effectively inhibits the activation and proliferation of microglia by suppressing the p38 MAPK pathway^96^. Furthermore, under pathological conditions, the abnormal accumulation of α-synuclein can activate immune cells such as microglia and astrocytes. Research has indicated that α-synuclein colocalizes with P2X7Rs in activated microglia, increasing P2X7R transcription^105^. α-synuclein oligomers activated microglia via the P2X7R/PI3K/Akt signaling pathway, which leads to the release of exosomes. This process subsequently mediates excessive microglial neurotoxicity, resulting in neuronal death^106^.

### P2X7R and Mitochondrial Dysfunction

Mitochondrial dysfunction is strongly linked to a range of neurodegenerative diseases. Current studies suggest that genetic and environmental factors that induce oxidative stress and mitochondrial dysfunction in dopaminergic neurons are important contributors to the pathological processes of PD^107,108^. Several identified PD risk genes, including *PARKIN*, *PINK1*, and *DJ-1*, are closely involved in the maintenance of mitochondrial function^109–111^. Additionally, postmortem examinations of PD patients have revealed significant mitochondrial dysfunction in the substantia nigra^112^.

It is generally understood that the activation of P2X7R leads to an increase in the intracellular calcium level, which subsequently impacts the mitochondrial membrane potential. High concentrations of calcium can trigger the opening of the mitochondrial permeability transition pore, causing a decrease in the mitochondrial membrane potential and impairing normal mitochondrial function^113^. Moreover, mitochondrial dysfunction can result in insufficient energy supply and increased oxidative stress, which may in turn influence the expression and activity of P2X7R, further exacerbating cellular damage^114^. In a mouse model overexpressing A53T α-synuclein, the accumulation of α-synuclein within mitochondria was found to increase mitophagy and neuronal death^115^. One of the key mechanisms underlying α-synuclein-induced mitochondrial dysfunction and increased free radical production is the activation of the purinergic receptor P2X7R^116^. Interestingly, research has indicated that in a 6-OHDA animal model, the non-selective purinergic receptor inhibitor pyridoxal 5′-phosphate-6-azo-phe-2017nyl-2,4-disulfonate (PPADS) and the P2X7R inhibitor BBG provided protection to the substantia nigra against the loss of mitochondrial integrity and dysfunction^117^. These studies suggested that P2X7R plays a crucial role in mitochondrial dysfunction during the onset and progression of PD.

### P2X7R and α-synuclein

α-synuclein was first described in the literature in 1994, and its three variants (A53T, A30P, and E46K) are closely associated with the onset of PD. In fact, α-synuclein is regarded as a major pathological hallmark of PD, as it is the primary component of Lewy bodies^118,119^.

P2X7R, the ATP-sensitive cation channel, is linked to the misfolding of and the oxidative stress induced by α-synuclein. First, in the pathological context of PD, there is a presence of misfolded α-synuclein. The misfolding of fibrillary or higher-order oligomers can cause toxicity to functionally important proteins, including the dopamine transporter, while also affecting processes in the substantia nigra and leading to mitochondrial dysfunction. This is closely related to reductions in energy production and cell death triggered by P2X7R^120^. Furthermore, research has shown that α-synuclein is present in both mitochondria and the extracellular space, where it can directly interact with the transmembrane domain of P2X7R, leading to alterations in P2X7R activity^113^. One study demonstrated the stimulatory effect of α-synuclein on P2X7R and significant mobilization of Ca^2+^ in an SH-SY5Y neuronal cell model^116^. Additionally, in microglia, P2X7R is directly involved in the exacerbation of oxidative stress via stimulation from extracellular α-synuclein^105^. Finally, mitochondrial depolarization typically results in decreased mitochondrial respiration, disruption of mitochondrial complex I activity, and consequently increased free radical production, which is closely related to the activation of α-synuclein^120–122^. The effects of α-synuclein largely depend on the activation of P2X7R, which induces the recruitment of Panx-1 and facilitates the formation of pores that are permeable to large molecules up to 900 Da^116^. These findings indicate that P2X7R plays a crucial role in the pathological processes mediated by α-synuclein.

## P2X7R Antagonists in PD Research

Based on the future potential of P2X7R, many companies such as Janssen Pharmaceutica NV, Acetelion Pharmaceuticals LTD., Renovis Inc., Kelly Michael G, Kincaid Jhon, Merck Patent GMBH, have invested a great deal of effort in using it as one of the most researched subtype in drug development for different diseases^123^. Numerous potent and selective antagonists, primarily allosteric antagonists, have been reported^124^. However, improved P2X7R antagonists with favorable pharmacokinetic properties have emerged and should be prioritized^123–125^. Like many allosteric regulators, some P2X7R antagonists exhibit species-specific differences. The earliest clinical trials evaluated P2X7R antagonists, such as AZD9056, for the treatment of rheumatoid arthritis, but their effectiveness for this indication was unconvincing^126^. The P2X7R antagonists currently available belong to a range of compound categories, some of which demonstrated high brain penetrance. Clinical assessments have shown that JNJ-54175446 can be used for treating major depressive disorder and bipolar disorder^127^. Furthermore, positron emission tomography PET tracers labeled with 11 C and 18 F have been developed^128^. Similarly P2X7 may serve as a drug target in PD diagnosis and treatment. A recent Mendelian study screening 313 differentially expressed genes that can be targeted in PD found that P2X7R was highly expressed in PD tissues and increased with increasing Braak stage^129^. This study provides genetic support for the potential therapeutic properties of targeting P2RX7, which will help P2X7R develop targets that can be used pharmacologically to treat PD.

Research on P2X7R in PD is currently focused primarily on preclinical stages (Table 1) with a strong emphasis on Brilliant Blue G (BBG), which is considered to be a potent competitive antagonist of the P2X7 receptor, however, this compound also targets P2X4, P2X2, rat P2Y1, and P2Y2 receptors, as well as voltage-gated sodium channels^130,131^. BBG was first launched in Europe in August 2010 and has gained widespread use in approximately 74 countries. Efforts are ongoing to develop various new derivatives of BBG for clinical application. Studies have shown that BBG preferentially binds to P2X7R^132^. It can block P2X7R on cells at nanomolar concentrations. Additionally, as previously mentioned, BBG can inhibit voltage-gated sodium channels in mouse neurons, thereby blocking P2X7R in mice^130^. Currently, it is primarily used as a surgical adjunct due to its excellent water solubility, allowing rapid penetration across the blood-brain barrier^133^. Initial studies on BBG in PD patients revealed that it could reduce contralateral rotations in rats induced by 6-OHDA. Furthermore, by reducing the ex pression of cytochrome c, caspase-9, and caspase-3 in the striatum, BBG was shown to mitigate mitochondrial-related apoptosis and exert a neuroprotective effect^97,108^. Additionally, BBG can reduce the loss of tyrosine hydroxylase-immunoreactive neurons in the substantia nigra by inhibiting the p38 MAPK pathway^96^. While L-DOPA is a classic clinical drug for treating PD, its long-term use may lead to the development of dyskinesia. Analu A. Fonteles et al. reported that continuous injection of BBG for 7 or 14 days significantly improved L-DOPA-induced dyskinesia, involving the regulation of dopamine D1 and D2 receptors in the striatum and substantia nigra^134^. These findings suggest that P2X7 antagonists may serve as new anti-dyskinetic drugs. Additionally, A-438079 has also been used as an antagonist in PD research, could prevent the depletion of striatal DA stores caused by 6-OHDA^97^. For cellular experiments, PPADS, a P2X7R antagonist, slowed the abnormal influx of calcium ions induced by α-synuclein and thus reduced the cytotoxicity to SHY5Y cells^135^ Another a New dual receptor antagonist named “9 g”, targeting P2X7R and NMDA could exert physiological effects at low concentrations which was designed by Karoutzou and colleagues^136^. And Kunitz-type peptides from sea anemones almost completely inhibiting the ATP-induced uptake of YO-PRO-1 dye in Neuro-2a cells through P2X7, which shows the application of a P2X7R inhibitor can attenuate the progress of a neurodegenerative process^137^. In summary, these findings indicated that developing P2X7R inhibitors may represent a new strategy for treating PD.

**Table 1:** Application of P2X7R antagonists in PD

Compounds	Mode of administration	Changes in P2X7R	Model/ Cell	Result	Year
					publised
A-438079	30 mg/kg	/	6-OHDA rat model	Significantly prevented the 6-OHDA-induced depletion of striatal DA stores	2010
KN-62、oxATP	oxATP 100 μM、 KN-62 1 μM	/	THP-1	Reduction of THP-1-induced inflammation by bzATP stimulation	2011
BBG	10 μM	/	Primary astrocytes	Promotion of astroglial TNFa secretion	2011
BBG	BBG (10 and 100 nM) /	/		Protected against MPTP and rotenone induced toxicityn the LDH assay	2011
AZ10606120	AZ10606120 (10 and 100 nM)	up-regulated	PC12 Cell		
BBG	BBG, 45 mg/kg, ip, once every two days for 2 weeks；BBG, 100 nM in cell expriments	/	6-OHD rat model；H-SY5Y	Improving memory impairment. Reduced glial cell activation	2014
BBG	1 mM	Increased the transcript levels of P2X7R after treatment with WT or A53T α-Syn	BV2, primary microglia	Extracellular α-Syn stimulates microglial P2X7 receptor with increased PI3K/AKT activation and oxidative stress	2015
BBG	50 mg/kg 1 h prior to administering the LPS injectio for 15 days	Increased the transcript levels of P2X7R after treatment with LPS	LPS-induced Parkinson disease	Reduced activation of the microglia and the loss of nigral DA neurons	2017
BBG	50 mg/kg daily for 7 days	Decreased expression of P2X7R	6-OHDA rat model	P2X7R antagonist BBG reverses the depletion of dopaminergic neurons	2017
PPADS AZ 11645373	AZ 11645373 (10 μM)	/	SH-SY5Y	Prevented abnormal calcium influx induced by α-synuclein	2017
BBG	50 mg/kg daily for 7 days	Decreased expression of P2X7R	6-OHDA rat model	P2X7R antagonist BBG reverses the depletion of dopaminergic neurons	2017
BBG	5, 25, 50 and 75 mg/kg daily for 7 days	Increased expression of P2X7R after treatment with 6-OHDA	6-OHDA rat model	Present neuroregenerative or neuroprotective effects	2019
BBG	BBG (2 nmol) intracerebrally at the same time 1 h before	/	6-OHDA rat model	Having stronger effect on mitochondrial biogenesis	2020
A-740003	100 nM	/	SN4741 cell	P2X7 receptor activation does not mediate ATP-stimulated Erk phosphorylation	2021

## Conclusions and Prospects

**Fig. 4:**
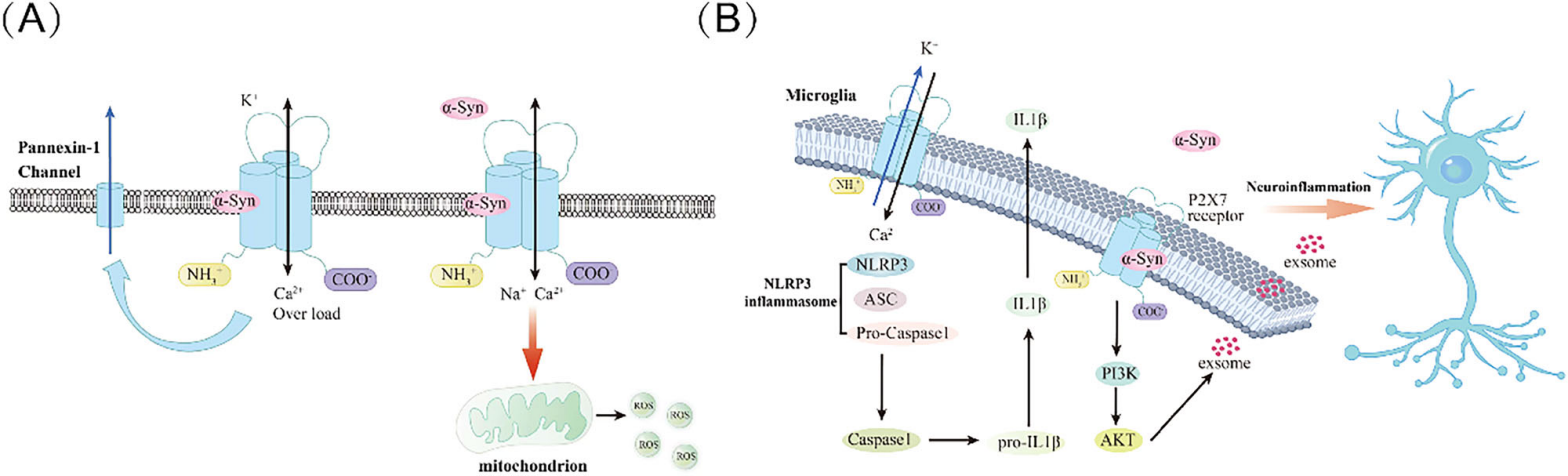
Role of P2X7R in PD. **A** There is a α-synuclein binding site on P2X7R. Throughout the development of PD, the interaction between α-synuclein and P2X7R can lead to intracellular Ca^2+^ overload. P2X7R also mediates the oxidative stress and mitochondrial dysfunction caused by α-synuclein. **B** When ATP binds to P2X7R on the cell membrane, it facilitates the assembly of inflammasomes, affecting the secretion of mature IL-1β. Additionally, α-synuclein oligomers can activate the PI3K/AKT pathway through the microglial P2X7R, increasing the release of CTSL-containing exosomes, which exacerbates neuroinflammation and contributes to neuronal death in PD.

In this review, we emphasized the function and structure of P2X7R, a member of the P2X family involved in purinergic receptor signaling. P2X7R is expressed predominantly in microglia and astrocytes, and further investigation is needed regarding its expression in neurons. Research has indicated that expression levels of P2X7R are elevated in patients with PD, particularly in the peripheral system during the early stages of the disease. These findings suggested that peripheral P2X7R may play a role in the early phases of PD. Additionally, preclinical studies have linked P2X7R with neuroinflammation and mitochondrial dysfunction, both of which are significant factors in the pathogenesis of PD, especially concerning the disease’s hallmark pathology, α-synuclein (Fig. 4). Therefore, P2X7 may serve as a promising target for the diagnosis and treatment of PD.

There are currently few antagonists that target P2X7R for the treatment of PD. However, selective P2X7R antagonists such as BBG and A-438079 have shown promising therapeutic effects in preclinical studies for PD. Notably, inhibiting P2X7R has been found to improve dyskinesia caused by long-term L-DOPA therapy. Furthermore, as the structure of P2X7R continues to be elucidated, there is ample reason to believe that antagonists targeting P2X7R could be developed into effective clinical treatments for PD.

## Data Availability

No datasets were generated or analysed during the current study.
